# Recent Advances in Pharmacological Management of Autism: A Narrative Review

**DOI:** 10.7759/cureus.101053

**Published:** 2026-01-07

**Authors:** Pallavi Abhilasha, Monika Sharma

**Affiliations:** 1 Psychiatry, Christian Medical College and Hospital, Ludhiana, IND; 2 Pediatric Critical Care, Christian Medical College and Hospital, Ludhiana, IND

**Keywords:** diagnostics, interventions, neurodevelopmental, therapeutics, treatment strategies

## Abstract

Autism Spectrum Disorders (ASD) are a highly heterogeneous neurodevelopmental conditions defined by impairments in social communication, reciprocal interaction, and the presence of restricted or repetitive behaviors. While its “spectrum” nature highlights variability in symptom severity and presentation, ASD often coexists with conditions like attention deficit hyperactivity disorder (ADHD), anxiety, depression, epilepsy, digestive, metabolic, and immune disorders. No pharmacological treatments currently address the core deficits of ASD; instead, behavioral and educational interventions remain central. Medications, including US Food and Drug Administration (FDA)‑approved antipsychotics such as risperidone and aripiprazole, are used to manage comorbid irritability and aggression, stimulants and non‑stimulants (e.g., methylphenidate, atomoxetine, clonidine, guanfacine) to treat ADHD-like symptoms, and melatonin for sleep disturbances. Other off‑label agents like selective serotonin reuptake inhibitor (SSRIs) for anxiety/obsessive compulsive disorder (OCD), anticonvulsants or mood stabilizers for mood dysregulation. Emerging compounds such as intranasal oxytocin and N‑acetylcysteine are under investigation but have not yet been formally approved. The future of ASD care hinges on the development of objective, biologically based diagnostic tools ranging from EEG and neuroimaging biomarkers to proteomic and metabolomic panels that could enable early, precise identification and guide personalized treatment strategies.

## Introduction and background

Introduction 

Autism spectrum disorders (ASD) encompass a diverse range of neurodevelopmental conditions that significantly impact an individual's social interactions, communication abilities, and restrictive behavioural patterns. These disorders typically manifest early in childhood and can persist throughout a person's life, although the symptoms and their severity can vary widely among individuals. The core features of ASD often include challenges in social communication and interaction, such as difficulty understanding and using nonverbal cues, including facial expressions and gestures, as well as trouble forming and maintaining relationships. Individuals with ASD may also exhibit repetitive behaviours or restricted interests, showing a preference for routines and experiencing distress with changes in their environment or schedule [1].

The term "spectrum" in ASD emphasises the variability in symptoms and the wide range of abilities and characteristics that can occur. Some individuals with ASD may have relatively mild challenges and be able to live independently, while others may require significant support with daily living skills and communication [2]. Research into ASD suggests that both genetic and environmental factors influence its development. The exact cause leading to autism is still unknown [3]. ASD are indeed more prevalent in boys compared to girls, with current estimates suggesting a ratio of approximately 4:1. This means that boys are diagnosed with ASD about four times more frequently than girls. Additionally, the prevalence of ASD have been increasing over the past few decades, and as of recent estimates, it is found in approximately one in 59 children [4].

There is a significant shift in how autism is understood and approached. The move from viewing autism purely as a medical condition in need of a cure to recognising it as a part of human diversity under the neurodiversity paradigm represents a broader societal change. This shift emphasizes the importance of acceptance, inclusion, and understanding of autistic individuals as part of the natural variation in human neurology. Pharmacological treatment in ASD help manage associated symptoms such as irritability, aggression, anxiety, and hyperactivity that interfere with therapy participation. Although behavioural interventions are central to care, their effectiveness may be limited in severe cases. Current medications mainly target secondary symptoms and have minimal effect on core social and communication deficits. Thus, there is a need for novel, mechanism-based drugs to provide safer and more individualized treatment options.

In recent years, particularly post 2020, autism research has shifted from descriptive behavioural frameworks toward elucidating underlying neurobiological mechanisms. Advances in understanding excitatory-inhibitory imbalance, gamma-aminobutyric acid (GABAergic) and oxytocinergic dysfunction, and neuroinflammatory pathways have catalysed the development of mechanism-based pharmacological interventions. Given the rapid evolution of this field and the lack of comprehensive medical treatments targeting core ASD features, this narrative review is timely and necessary. It synthesises emerging evidence on novel drug targets, evaluates their translational relevance, and contextualises pharmacological advances within the broader movement toward personalised, neurobiologically informed treatment in ASD.

## Review

Materials and methods

Search Strategy

Two authors independently searched MEDLINE (PubMed) and Google Scholar from inception till November 2025. A Boolean search was conducted using a combination of the following MeSH and text terms: “Autism”, “recent advances”, “pharmacological management”, “new drugs”, and “EEG”.

The search was limited to English-language studies conducted in children and focused specifically on recent advances in the pharmacological management of ASD. Although autism management is multifaceted and also involves important domains such as genetic testing, educational integration, employment support, and social assimilation, these aspects were intentionally excluded, as the present narrative review was restricted to pharmacological interventions.

Data Extraction

There were a total of 10,826 articles related to recent advances in the pharmacological management of ASD on the PubMed database. A combination of search terms, including “ASD and pharmacological management,” yielded 3821 results. After screening for titles and abstracts, a total of 17 articles were included for our review. A total of two articles were included for biofeedback and neuromodulation in ASD, and two articles on EEG and ASD were included for this study.

Drug Interventions

The body of research around pharmacological interventions for ASD has expanded significantly in recent years, reflecting the versatility and nuanced understanding of how these drugs can impact the bio-physiology of individuals with ASD. The interventions provided by these drugs are designed to target specific symptoms or underlying physiological processes associated with ASD, which can vary widely among individuals. As a result, the effectiveness and approach to pharmacotherapy can differ depending on whether the signs and symptoms are fully developed or still emerging. This individualized approach highlights that autism is a highly heterogeneous condition, where symptoms and biological factors differ greatly among individuals. Therefore, treatment strategies must remain flexible, evolving with new research findings and the patient’s changing needs. Continuous research and clinical adaptation are essential to ensure that therapeutic interventions are both effective and personalized for every person with ASD.

Pharmacotherapy is viewed not just to manage symptoms but also as a potential tool for rehabilitation, aiming to influence the underlying biological mechanisms of ASD in a way that supports overall well-being and integration. This area of study remains a dynamic field, with continuous developments contributing to more tailored and effective interventions. Medications commonly used for the management of ASD include antipsychotics, antidepressants, anxiolytics, and stimulants. The two antipsychotics which have been FDA approved for the aggressive and stereotypic behaviour in children are risperidone and aripiprazole (Table 1).

**Table 1:** ASD with behavioural issues This table presents first-line medications commonly used to manage significant behavioural challenges in individuals with autism spectrum disorders (ASD) [5]. Risperidone and aripiprazole are effective but require close monitoring due to potential side effects. These include sedation, weight gain, and serious metabolic or neurological risks. Clinicians should balance benefits with safety and adjust treatment based on individual response.

First-line medication	Side effects
Risperidone, Aripiprazole	Excessive sedation, weight gain, metabolic side effects, dyskinesia, neuroleptic malignant syndrome

Selective serotonin reuptake inhibitors (SSRIs) may help manage anxiety and depressive symptoms in individuals with ASD. These medications may also be associated with potential side effects and risks, including weight gain, behavioural changes, and metabolic issues [5]. Stimulant medications, including methylphenidate and amphetamines, are commonly prescribed to manage co-occurring symptoms of hyperactivity, impulsivity, and inattention, particularly when attention deficit hyperactivity disorder (ADHD) is diagnosed alongside ASD. For mood-related concerns, SSRIs such as fluoxetine and sertraline are frequently used to reduce anxiety and depressive symptoms. In more severe cases of anxiety or acute behavioural dysregulation, short-term administration of benzodiazepines like clonazepam or lorazepam may be employed with caution, given their potential for sedation, dependence, and paradoxical effects in individuals with ASD. Repetitive behaviour, which is a sign of autism, is significantly reduced with fluoxetine and citalopram [6]. Bupropion can be given if the child is having ADHD along with ASD symptoms. Common side effects include insomnia, agitation, headache, dry mouth, tremor, and gastrointestinal discomfort. Of particular concern is its dose-dependent risk of lowering the seizure threshold, which may precipitate seizures, especially in individuals with predisposing factors. Other less common adverse effects include anxiety exacerbation and, rarely, suicidal ideation, particularly in younger patients. Bupropion is contraindicated in individuals with a history of seizure disorder, bulimia nervosa, or anorexia nervosa, as these conditions further increase seizure risk. In children with comorbid seizure disorder, bupropion can lower the seizure threshold and should not be used [7]. Before initiating any pharmacological treatment in children with ASD, it is crucial to conduct a comprehensive diagnostic assessment, develop an individualized treatment plan, and ensure consistent monitoring and close follow-up. Patient’s needs, severity of symptoms, and side effects must be considered before the initiation of any medication. Behavioural interventions, along with educational support, should be used in tandem to achieve therapeutic outcomes [2]. Table 2 outlines pharmacologic treatment options for individuals with ASD comorbid with ADHD. 

**Table 2:** Pharmacologic treatment options for individuals with autism spectrum disorders (ASD) comorbid with attention deficit hyperactivity disorder (ADHD) Medication choice should be guided by the presence of comorbid symptoms such as anxiety, sleep disturbances, or tics. Non-stimulants are preferred when stimulants are contraindicated or poorly tolerated. Tailored approaches improve outcomes by addressing both core and associated behavioural challenges [7].

Decision point	Treatment options	When to choose
First-line therapy: Significant emotional dysregulation, behavioural problems beyond core ADHD, or medical contraindications to stimulants	Guanfacine, clonidine, atomoxetine	Choose these when common stimulant treatments may pose risks or are less suitable
Yes, co-occurring anxiety, sleep problems, tics?	If any sleep issues are present along with anxiety, give clonidine. If anxiety + sleep disturbances and tics are present, try atomoxetine or viloxazine. For tics, guanfacine or clonidine	Tailor choice based on specific coexisting symptoms. Requires targeted management.
Otherwise, no contraindications or comorbidities	Stimulants: Methylphenidate, dextroamphetamine	First-line standard medications where there are no major additional concerns

In addition to psychotropic medications, several non-psychotropic agents are also employed in the management of ASD, targeting associated symptoms rather than core psychiatric features. Naltrexone, an opioid receptor antagonist, is occasionally used to reduce self-injurious behaviours and aggression by modulating the brain’s endogenous opioid system. Sleep disturbances, which are common in individuals with ASD, are often managed with melatonin, a hormone that helps regulate circadian rhythms and improve sleep quality. Clonidine, an antihypertensive agent with sedative properties, is sometimes prescribed to address hyperactivity and impulsivity, offering a calming effect. Similarly, guanfacine, another alpha-2 adrenergic agonist, is used to modulate norepinephrine activity, contributing to reductions in hyperactivity and behavioural dysregulation. In select cases, memantine an N-methyl-D-aspartate (NMDA) receptor antagonist may be used to manage repetitive behaviours and aggression by influencing glutamatergic neurotransmission (Table 3) [6].

**Table 3:** Sleep medications in autism This table outlines pharmacological treatments commonly used for managing sleep disturbances, particularly in children or individuals with neurodevelopmental conditions such as autism spectrum disorders (ASD) [6]. Treatments are categorized by treatment stage (first-line vs. second-line), with details on the medication, typical starting dose, key therapeutic effects, and potential side effects. Dosages should be tailored to individual patient needs under clinical supervision

Treatment stage	Medication	Typical starting dose	Key effects	Side effects
First line	Melatonin	3 mg, 30 minutes before bedtime, can be given up to 10 mg if required.	Reduces sleep onset latency, can improve total sleep time	Drowsiness, headache
Second line	Clonidine	Start 0.05-0.1 mg	Improves sleep initiation and maintenance latency	Sedation, hypotension, bradycardia
	Guanfacine	Start 0.5 to 1 mg	Used when clonidine is not tolerated, for maintenance in some ASD cases	Daytime sleepiness, orthostatic hypotension

Intranasal oxytocin has indeed shown promise as a therapeutic option for children with ASD, particularly in areas related to social functioning. Research suggests that oxytocin can enhance emotional processing, improve eye contact, and address some of the social impairments commonly associated with ASD. These findings highlight the potential of oxytocin as part of a broader treatment plan aimed at improving social interactions and emotional recognition in children with ASD, offering a targeted approach to some of the core challenges they face [8].

Bumetanide is a loop diuretic that inhibits the sodium-potassium-chloride Cotransporter 1 (NKCC1) cotransporter, reducing intracellular chloride levels in neurons. This shift helps restore the inhibitory action of GABA, which is often altered in autism due to elevated intracellular chloride. By promoting more typical GABAergic signalling, bumetanide may improve neural network balance, potentially reducing core autism-related symptoms such as sensory dysregulation, irritability, and social communication difficulties. The randomized waitlist-control study involving 15 children with autism found that bumetanide led to clinically meaningful improvements in a subset of participants, with four of nine children completing the study showing notable symptom reduction according to the reports from their parents. Despite several dropouts due to behavioural issues or tolerability, the findings suggest that bumetanide may benefit select children with ASD. The study adds supportive evidence but highlights variability in treatment response [9]. Another study investigated whether resting-state EEG patterns and baseline clinical severity could predict which children with autism would show behavioural improvement when treated with bumetanide. The findings showed that specific EEG signatures, particularly those reflecting excitatory-inhibitory imbalance, were associated with better clinical response, suggesting that neurophysiological markers may help identify likely treatment responders. This supports the idea of personalized, biomarker-guided treatment approaches in ASD [10].

A Phase II randomized, double-blind, placebo-controlled Autism Innovative Medicine Studies-2-Trials (AIMS-2-TRIALS) outlines the protocol for evaluating arbaclofen as a potential treatment to improve social functioning in children and adolescents with ASD. Arbaclofen, a selective GABA-B receptor agonist, is hypothesized to reduce social difficulties by modulating the excitatory-inhibitory imbalance in the autistic brain. The trial aims to generate high-quality evidence on the efficacy, safety, and tolerability of the treatment [11].

Naviaux’s review outlines the Cell Danger Response (CDR) theory, which proposes that abnormalities in purinergic signaling contribute to core autism symptoms. The paper discusses how anti-purinergic agents, particularly suramin, may normalise cellular communication, reduce neuroinflammation, and improve social and metabolic functioning. It highlights early clinical findings showing promising but preliminary benefits, while emphasizing the need for larger, controlled trials to confirm safety and efficacy [12].

Vasopressin Receptor Antagonists

László et al. reviewed evidence that the neuropeptide vasopressin, which regulates social behaviour, stress responses, and emotional processing, may play a significant role in the neurobiology of autism. The authors described how disruptions in the vasopressin signalling, particularly via the V1a receptor pathways, could contribute to social communication deficits seen in ASD. They concluded that targeting the vasopressin system, including intranasal vasopressin or V1a-receptor modulation, represented a promising but still experimental therapeutic approach, likely beneficial only for specific subgroups of individuals with autism [13].

Another review highlighted that individuals with ASD, especially those with gastrointestinal symptoms, often showed significant alterations in their gut microbiota. It explained that the gut-brain axis allowed microbial metabolites and immune signals to influence brain development and behaviour. Evidence from both human studies and animal models suggests that gut dysbiosis may contribute to ASD-related symptoms. The authors proposed that modifying the gut microbiota through probiotics, dietary changes, antibiotics, or fecal microbiota transplantation could be a promising therapeutic strategy. However, they emphasized that findings are inconsistent, and more well-designed clinical studies are needed to confirm the effectiveness and safety of microbiota-based treatments [14].

Biofeedback and Neuromodulation

Biofeedback and neuromodulation represent recent and emerging therapeutic strategies for managing symptoms of ASD. The former involves training individuals to gain voluntary control over involuntary physiological functions such as brainwave patterns, muscle activity, and heart rate by providing real-time monitoring and feedback. This in turn enhances focus, decreases anxiety levels, and promotes better emotional self-regulation [15].

Neuromodulation techniques include transcranial magnetic stimulation (TMS) and transcranial direct current stimulation (tDCS). These newer technologies aim to influence brain function through non-invasive external stimulation. TMS uses magnetic pulses to stimulate targeted brain regions, whereas tDCS delivers low-intensity electrical currents to alter neuronal excitability. Exciting potential exists in improving social interaction and communication skills, as well as decreasing repetitive or stereotyped behaviours in individuals with ASD [15]. TMS decreases the power of gamma activity and increases the difference between gamma responses to target and non-target stimuli, thereby improving executive function skills related to self-monitoring behaviour [16]. These approaches are still nascent in the context of autism treatment. While they offer potential, particularly for improving core symptoms like social deficits, repetitive behaviours, and cognitive functions, there is a need for more data and clinical research to determine their generalisability on a larger scale.

EEG and ASD

There is a growing area of research that focuses on EEG features in ASD, particularly on background abnormalities and interictal epileptiform discharges (IEDs). While there is no definitive evidence on the diagnostic, prognostic, or therapeutic role of EEG in ASD, these features hold potential in understanding the heterogeneity of the disorder and in refining the identification of subgroups within the autism spectrum. IEDs are bursts of abnormal electrical activity in the brain that can occur between seizures but are also found in individuals with no clinical seizures, as seen in many with autism. Studies have found that paroxysmal slow-wave IEDs (PS-IEDs) occur more frequently in individuals with ASD, with rates ranging from 5% to 46%, whereas they are rare in healthy populations (1-4%). These abnormalities are often considered nonspecific signs of cortical dysfunction and may not always indicate clinical epilepsy. However, they may play a role in shaping certain neurodevelopmental characteristics, contributing to the autism phenotype. PS-IEDs have been found in approximately 30% of individuals with ASD who show no clinical evidence of epilepsy. This indicates a possible link between abnormal cortical activity and the cognitive, behavioural, and social impairments seen in ASD, even in the absence of seizures. The heterogeneity of autism presents challenges for both diagnosis and treatment. EEG features could help reduce this variability by identifying specific subgroups of ASD individuals, especially those with underlying electrical brain abnormalities. These findings suggest that cortical dysfunction, even when not associated with overt seizures, may contribute to the neurological features of ASD, potentially influencing behaviours such as social communication deficits and repetitive behaviours. In the study conducted by Santarone et al., PS-IEDs are proposed as a potential neurophysiological marker for ASD, reinforcing the idea that autism may stem from disruptions in the neural circuitry of cortical and subcortical regions [17]. Hence, PS-IEDS represent a promising avenue for reducing the heterogeneity in ASD and would help in developing more personalised treatment.

Limitations

Despite notable scientific advancements, several limitations continue to constrain ASD research and pharmacological development. First, the heterogeneity of ASD presents a major challenge, as biological mechanisms, symptom profiles, and treatment responses vary widely across individuals. This variability limits the generalisability of research findings and underscores the need for more precise, biomarker-guided stratification. Second, evidence for many emerging pharmacological agents remains preliminary, often based on small sample sizes, short trial durations, or heterogeneous study designs. Larger, long-term randomised controlled trials are still needed to establish safety, efficacy, and target populations for therapies such as bumetanide, suramin, oxytocin, vasopressin modulators, and neuromodulation techniques.

A further limitation is the lack of longitudinal research tracking developmental trajectories across the lifespan, especially into adulthood. Limited focus on adults, particularly those diagnosed later in life, prevents the development of age-specific interventions and long-term management strategies. Additionally, comorbid conditions such as ADHD, anxiety, epilepsy, and gastrointestinal disturbances complicate diagnosis and treatment, yet remain insufficiently understood at the mechanistic level. While emerging tools such as genetic testing, EEG biomarkers, neuroimaging, and microbiome profiling offer diagnostic potential, they are not yet integrated into routine clinical practice due to cost, accessibility, and insufficient validation.

Finally, the gap between biomedical research priorities and the practical needs of autistic individuals remains a critical limitation. Many families emphasise the need for improved support systems, accessibility, and functional outcomes that extend beyond pharmacological symptom control. Addressing these gaps requires a more inclusive, lifespan-oriented research approach with active participation from autistic individuals to ensure that scientific advancements align with real-world priorities.

## Conclusions

ASD are complex neurodevelopmental condition arising from an interplay of genetic, neurological, and environmental factors. Its multifactorial nature makes both diagnosis and research challenging, though advances in genetics, neurobiology, and technology continue to deepen understanding and improve care. Genomic studies have identified both common and rare variants that contribute to ASD, shedding light on gene-environment interactions and epigenetic mechanisms. Parallel research on biomarkers is helping refine diagnosis, early detection, and individualised interventions. Current approaches increasingly combine pharmacological treatments with behavioural and educational therapies, aiming to enhance overall functioning and quality of life rather than focusing solely on symptom reduction.

Transforming ASD diagnosis and management requires collaboration among researchers, clinicians, and individuals with autism. Integrating autistic voices ensures that diagnostic frameworks are developmental, contextual, and culturally sensitive. Adopting a strengths-based perspective, emphasising abilities and talents, promotes empowerment and engagement in therapy. Finally, ethical and social considerations must guide scientific progress. The focus of research should not be on “curing” autism but on fostering acceptance, inclusion, and accessibility. Transparent communication, co-production of research, and prioritising practical interventions that enhance daily functioning can build trust and ensure that scientific advances genuinely benefit the autistic community.
